# Lead and Cadmium Levels in Residential Soils of Lagos and Ibadan, Nigeria

**DOI:** 10.5696/2156-9614-7-13.42

**Published:** 2017-03-29

**Authors:** Adebola A. Adeyi, Babafemi A. Babalola

**Affiliations:** Department of Chemistry, University of Ibadan, Ibadan

**Keywords:** soil, lead, cadmium, contamination, heavy metal, paint

## Abstract

**Background.:**

Lead and cadmium are components of paints sold in Nigeria. These heavy metals are associated with adverse neurological, cardiovascular and other human health effects.

**Objectives.:**

This study assesses the levels of lead and cadmium in topsoil of residential areas in Lagos and Ibadan potentially resulting from painting of buildings.

**Methods.:**

Samples were pre-treated prior to metal determination using atomic absorption spectrophotometry. Metal speciation was determined using modified Tessier sequential extraction. Soil characteristics were determined by standard methods. Soil contamination was assessed using contamination factor, geo-accumulation and pollution load indices. The United States Environmental Protection Agency integrated exposure uptake biokinetic (IEUBK) model was used to estimate and predict children's blood lead levels (BLL).

**Results.:**

Lead and cadmium concentrations in residential areas in Lagos and Ibadan ranged from 1.56–419 mg/kg and not detected–2.8 mg/kg, respectively. Metal contamination factor and pollution load index were highest at the Lagos low income settlement. Results of IEUBK modelling showed that the Lagos low income settlement had the highest probability density for children between 1–7 years of age with an estimated BLL of >10 μg/dL. This population made up less than 0.01% of those within this age range.

**Conclusions.:**

Lead and cadmium concentrations in soil around the residential buildings were higher than those in the control sample. Contamination factor and pollution load index showed significant contamination in average and low income settlements. These results suggested that there was accumulation of the metals in the soil, which can persist in the environment. This may pose serious health risks, especially to vulnerable groups such as children.

## Introduction

Soil contamination may result from the accumulation of heavy metals and metalloids such as lead, cadmium, mercury and nickel, resulting from industrialization, atmospheric deposition, waste disposal, petrochemical spillage, mine tailings, coal combustion residues, gasoline, paints and application of fertilizers and pesticides.^1^,^2^ Urban soil contamination by heavy metals is recognized as a form of point source pollution.^3^ Heavy metals released into the environment by human activities do not undergo microbial or chemical degradation, and thus can persist in soils long after their release.^4^,^5^ Elevated concentrations of heavy metals in soil is a possible threat to human health since humans can be exposed through accidental ingestion, direct contact, and inhalation of suspended dust.^6^

Paint flakes, chips, and dust are potential sources of lead and cadmium contamination in Nigeria.^3^,^7^ Paints contain lead and cadmium pigments which enhance durability and colour, respectively. Many countries have banned the use of lead in consumer products such as paints, and global elimination of lead in paints in all countries of the world is expected by the year 2020.^8^,^9^ In Nigeria, the agency with the responsibility for product standardization, the Standards Organisation of Nigeria, is working on developing new standards to reduce lead in paints by setting a benchmark of 90 ppm.^10^

A 2014 study on paints marketed in Nigeria showed that 83% of 59 samples had lead levels which exceeded the 600 ppm level recommended for new paints by the US Consumer Product and Safety Commission, while lead levels in these samples ranged from 22.5 to 74,175 mg/L.^11^ A recent study also showed that cadmium and lead concentrations in 174 water-based paints collected in Lagos and Ibadan ranged from 98–1946 μg/g and 173–3117 μg/g (dry weight), respectively. All the samples were above the permissible limits of 90 ppm of the US Consumer Product Safety Commission and 100 ppm of the European Union for lead and cadmium, respectively, in paints.^12^ Despite recent international efforts to reduce lead in paints in all nations by the Global Alliance to Eliminate Lead in Paint, paints containing high levels of lead are still available and sold in Nigeria.^11^,^13^ Because of toxicity concerns, cadmium pigments have been replaced by organic pigments such as pyrrole and the arylide family of pigments in many paints and varnishes, although paints containing high levels of cadmium are available in the country.^13^,^14^

**Abbreviations**
*BLL*: Blood lead level
*IEUBK*: Integrated exposure uptake biokinetic
*CF*: Contamination factor
*I_geo_*: Geo-accumulation index
*HNO_3_*: Nitric acid
*PLI*: Pollution load index

The presence of lead in paints and other consumer products in homes is associated with increased children's blood lead levels (BLL) and poisoning risk.^15–18^ Exposure of children to lead and cadmium in paint chips or dust can result from hand-to-mouth activity or from eating the chips directly.^14^ Compared with adults, a larger proportion of lead swallowed by children is absorbed in the gastrointestinal tract.^15^ In some places, BLLs in children are likely to be a consequence of ingesting lead from paints and other consumer products, rather than from automotive exhaust.^19^,^20^ Increased BLLs in urban children is most likely to be from contaminated household dust and ensuing hand contamination and mouthing behaviors.^21^

Elevated BLLs are a significant issue for public health.^22^ A number of studies have reported BLLs in the population of Nigeria. Elevated BLLs in children 1–6 years old were reported in Kaduna, a medium-sized city in the northern part of Nigeria.^20^ Mean BLL was found to be 10.6 μg/dL, and 2% of the children had BLLs greater than 30 μg/dL. Studies conducted in three cities in Nigeria with different levels of industrial pollution found the mean BLL in children to be 8.9±4.8 μg/dL, with a median value of 7.8 μg/dL, and a range of 1–52 μg/dL. About 25% of children had a BLL greater than 10 μg/dL, and levels of 48.50 ± 9.08 μg/dL were also reported.^23^,^24^

Excessive dietary intake of lead has been linked with cancers of the stomach, small intestine, large intestine, ovary, kidney, lungs, myeloma, lymphomas, and leukemia.^25^,^26^ Lead exposure causes health problems, including headache, vomiting, high blood pressure, fertility problems in men, miscarriage in women, developmental delay and damage to the nervous system in children.^27^,^28^ Lead is a particularly dangerous metal that has no biological role and negatively affects children in significant ways.^29^ Both postnatal and prenatal exposure to lead is associated with poor child neurodevelopment. Studies of postnatal exposure show evidence of long-term effects on childhood intelligence quotient, as well as attention and inhibition.^30–33^ Environmental cadmium exposure is associated with an increased risk of cancer and cardiovascular disease mortality among men, but not among women.^30^ Chronic exposure to cadmium may increase mortality and shorten life expectancy. This study assesses the levels and forms of lead and cadmium in soils of residential areas in Lagos and Ibadan, mainly contributed by painting the residential buildings. It evaluates soil contamination using the geo-accumulation index, contamination factor, pollution load index and correlation coefficient. Lead exposure levels were evaluated using the United States Environmental Protection Agency's integrated exposure uptake biokinetic (IEUBK) model to predict the risk associated with exposure of children ages 0–7 years.

## Methods

### Study Area

**Figure 1:**
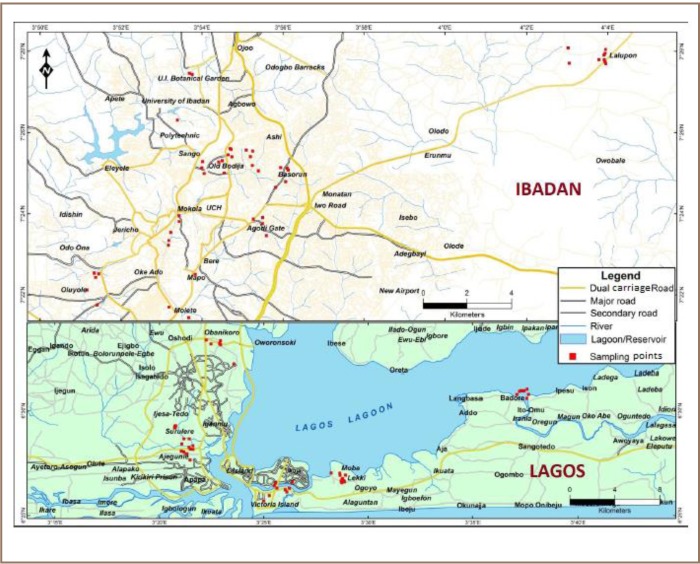
Map showing sampling points in Ibadan and Lagos

The study locations included residential areas in Ibadan and Lagos (*Figure 1*). Selection criteria were the settlement age, economic status, population and level of literacy of the residents. Each city was stratified into five settlements, based anecdotally on the age of the residential areas and income levels of the residents as follows: old high income, new high income, average income, low income and suburban settlements, based on Nigerian town planning standards. High, average and low income settlements were comprised of high, average and low income earners, while residents in the suburban settlement were poor with a low population and high level of illiteracy. The old and new settlements have been in existence for over 35 years and less than 10 years, respectively. Ten streets were purposively selected in each of the high, middle, low income and suburban settlements. In most cases, the residential buildings in the settlements were constructed with bricks made of cement, and remained unplastered in only a few cases.

### Soil Sample Collection

Soil samples were purposively collected from three painted buildings randomly selected in each street by scooping topsoil (0–15 cm) into a polythene bag with a plastic hand trowel. Samples were collected from the base of the painted buildings up to 0.5 m away. The sampling points around the selected buildings were 5 m to 8 m away from roads, garages and other sources of lead emission such as automobile emissions and potential petroleum product spills. A Garmin 72 model global positioning system unit was used to measure the coordinates of each sampling point (*Figure 1*). A total of 100 soil samples were collected around the selected residential buildings in the two cities, while two control samples were collected at the Botanical Garden, University of Ibadan, Ibadan, Nigeria. The samples were air dried at room temperature, crushed in a porcelain mortar and passed through a 2 mm sieve.

### Analytical Methods

The sieved and homogenized soil samples were analyzed for pH and electrical conductivity using a Hanna pH meter (H196107) and Hanna EC/TDS meter (H198129), respectively. Organic carbon was determined using the Walkley-Black titration method and soil particle size distribution by the hydrometer method.^34^,^35^ Samples for the determination of lead and cadmium were digested using 2 M nitric acid (HNO_3_) for 2 hours. The digested samples were filtered, diluted with deionized water and analyzed for total lead and cadmium using a Buck Scientific atomic absorption spectrophotometer (Buck Scientific model 210 VGP, USA). Appropriate quality assurance protocols were adopted to ensure reliable and reproducible results. The detection limits of the metals were calculated from the mean of the results of ten blank determinations inclusive of the outliers plus three times the standard deviation.^36^ A coefficient of variation of 10% duplicate analysis of the sample for lead and cadmium ranged from 0.63–10.9% and 0–14.6%, respectively.

The modified sequential extraction procedure of Tessier et al. was used to fractionate the soil lead and cadmium.^37^ The fractions include: (1) water soluble fraction: deionized water; (2) exchangeable fraction (readily available): 1.0 M magnesium chloride (MgCl_2_) at pH 7; (3) bound to carbonate: 1 M sodium acetate (CH_3_COONa) adjusted to pH 5 with acetic acid; (4) bound to iron-manganese oxides: 0.04 M hydroxylamine hydrochloride in 25% (v/v) acetic acid heated to 96±30^°^ C; (5) bound to organic matter: 0.02 M HNO_3_ and 30% hydrogen peroxide at pH 2 heated to 85±20° C, then 5 mL of 3.2 M ammonium acetate (20% (v/v)) was added; and (6) residual fraction: 4 M HNO_3_ heated at 800^°^ C. The supernatants obtained from each extraction step were acidified with 0.5 mL concentrated HNO_3_ and stored at 40^°^ C until further analysis using atomic absorption spectrophotometry.^38^ The fractionation of heavy metals is an important aspect of environmental studies to assess the amount of heavy metals that may be soluble, mobile and available to the terrestrial ecosystem, which total metal concentration alone cannot determine.

Mobility factors of the residential soils were calculated according to the expression by Kabala and Singh (*Equation 1*).^39^


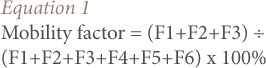


Where, F1 = water-soluble fraction, F2= exchangeable fraction, F3 = carbonate bound fraction, F4 = iron-manganese oxide bound fraction, F5 = organic matter bound fraction and F6 = residual fraction.

### Soil Contamination

To evaluate soil contamination of the study areas, three indices: contamination factor (CF) described by Hakanson, pollution load index (PLI) of Tomlinson et al. and geo-accumulation index (I_geo_) introduced by Muller were used to ascertain the extent of lead and cadmium contamination of the topsoil.^40–42^ CF was calculated using Equation 2.


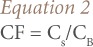


Where, C_s_ is the mean concentration of the target element in the samples and C_B_ is the baseline or background value (concentration of each metal in the control sample was used).^43^

A modified formula of CF was used as there is no established background value of heavy metals for Nigeria. Four categories adopted to describe the extent of contamination are CF <1: very low contamination, CF 1–3: low contamination, CF 3–6: moderate contamination, and CF >6: considerable contamination. Considerable contamination indicates that the metal concentration is 100% greater than that expected in the continental crust. Calculated as the geometric mean of lead and cadmium contamination factors of each sample, PLI is a composite descriptor of the combined lead and cadmium contamination of the residential soils.^44^ A value of one indicates only baseline levels of pollutants present and values above one indicate progressive contamination of the soil.^45^ The I_geo_ was calculated using Equation 3.


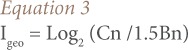


where, Cn is the concentration of the element n and Bn is the geochemical background value; Bn is 20 and 0.3 for lead and cadmium, respectively.

The factor 1.5 was incorporated to account for possible variation in the background data due to lithological effects. I_geo_ values were then categorized as <0: uncontaminated, 0–1: uncontaminated-moderately contaminated, 1.00–1.99: moderately contaminated, 2.00–2.99: more contaminated and 3.00–3.99: highly contaminated.

### Statistical Analysis

The data obtained (i.e., soil properties and metal concentrations) were subjected to descriptive statistical analysis. Analysis of variance (ANOVA) was used to determine whether there were significant variations in the concentrations of lead and cadmium in the soils and if the concentrations were significantly higher (p < 0.05) than the background level in order to ascertain the possibility of painting of the residential buildings as a potential source of lead and cadmium contamination. The correlation coefficient was used to determine whether there were significant relationships between lead and cadmium concentrations and soil physicochemical properties. The statistical analysis was performed using Statistical Package for Social Sciences (SPSS) version 16.0.

### Risk Assessment

The Windows-based version of the IEUBK model for lead in children (IEUBKwin v1.1 build 11) was used to estimate and predict the risk of lead exposure in children aged 0–7 years using the default value in the model, except for the lead concentrations obtained in this study.^46^

## Results

**Figure 2:**
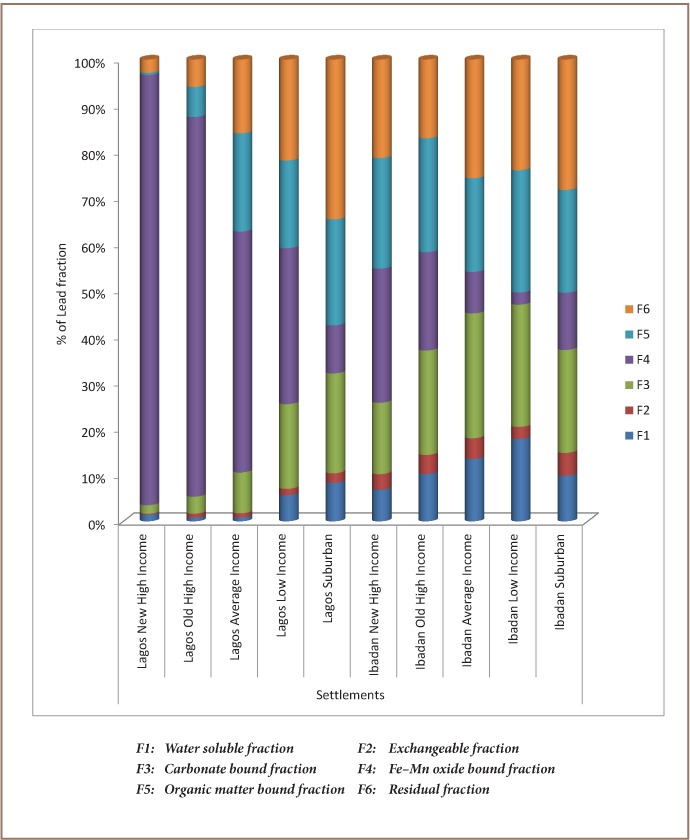
Fractionation of soil lead by settlement type

**Figure 3:**
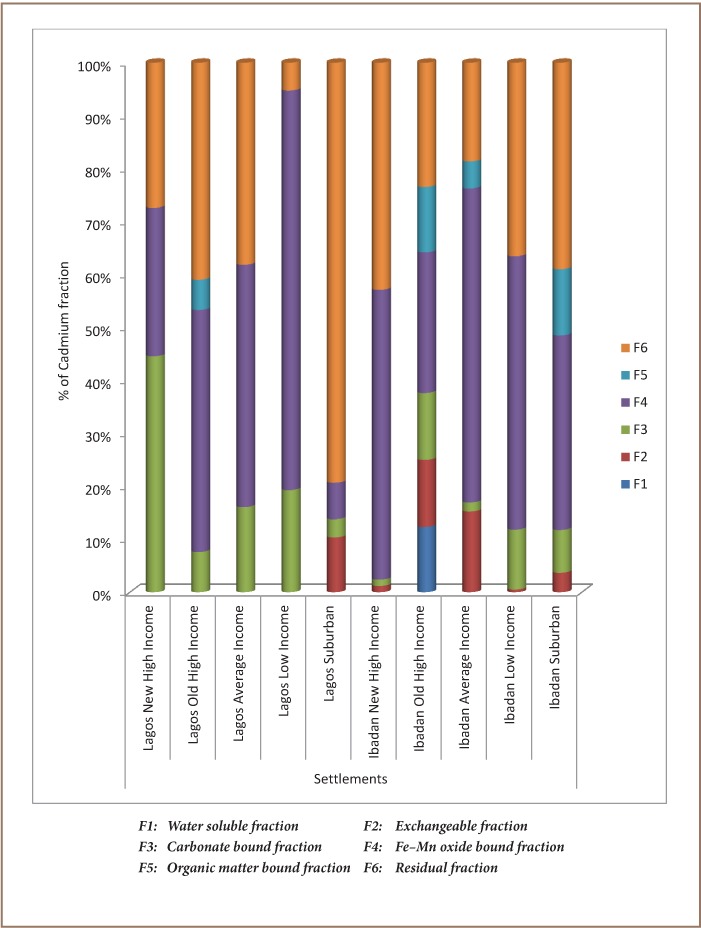
Fractionation of soil cadmium by settlement type

Table 1 shows the levels of the physicochemical properties, lead and cadmium in soil of the residential areas and control samples. The results of sequential extraction of lead and cadmium in the soil samples collected around the selected buildings in residential settlements in Lagos and Ibadan are illustrated in Figures 2 and 3.

**Table 1:**
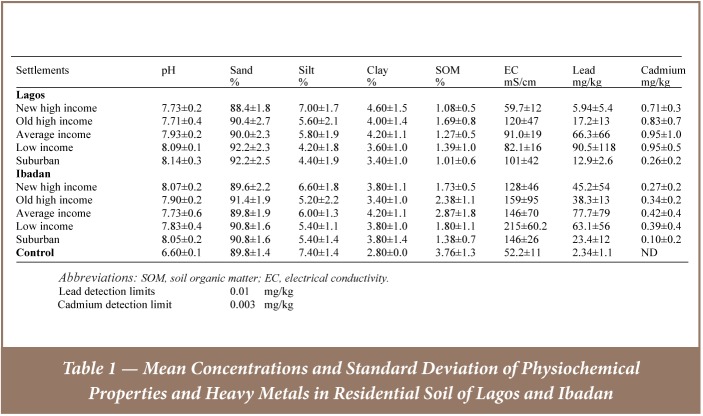
Mean Concentrations and Standard Deviation of Physiochemical Properties and Heavy Metals in Residential Soil of Lagos and Ibadan

### Physicochemical Properties and Heavy Metals in Soil of the Residential Settlements in Lagos and Ibadan, Nigeria

The soil of the residential areas in Ibadan and Lagos were dominated by coarse particles of sand, with mean particle size distribution varying from 4.20 ± 0.6 to 7.00 ± 0.5% silt; 3.40 ± 0.3 to 4.60 ± 0.4% clay; and 88.4 ± 0.6 to 92.2 ± 0.8% sand. The dominance of coarse particles of sand indicates the potential leaching of the pollutants deposited on the topsoil, but the silt and clay contents of the soil support retention of the leached pollutants. The soil samples were slightly alkaline with pH ranging from 6.40–8.70. The Lagos suburban settlement had the highest average pH of 8.14±0.1, while the lowest, 7.71±0.4, was obtained in the old high income settlement in Lagos (*Table 1*). The average pH of the control samples was 6.60, which was lower than the average pH of the soil collected in all the settlements in both cities. The mean electrical conductivity values ranged from 59 ± 3.9 to 215 ± 19 μS/cm and the mean soil organic matter ranged from 1.01 ± 0.2 to 2.87± 0.6%. The electrical conductivity value did not exceed the critical value of 4000 μS/cm for identifying the soil as saline.^47^ The concentrations of lead and cadmium ranged from 1.56–419 mg/kg and not detected–2.80 mg/kg, respectively, in all the residential settlements. Mean lead and cadmium concentrations in all the settlements ranged from 5.94±5.4–90.5±118 mg/kg in the new high income and low income settlements in Lagos; and 0.10±0.2–0.95±1.0 mg/kg in Ibadan suburban and middle and low income settlements in Lagos. The mean lead and cadmium concentrations in the control samples were 2.34±1.1 mg/kg and 0.46±0.7 mg/kg, respectively. When compared with the control samples, there was evidence of lead contamination in all of the settlements in both cities as the concentrations were above the level in the control sample, while cadmium contamination was evident in all the settlements in Lagos, except the Lagos suburban settlement. There was little or no cadmium contamination in any of the settlements in Ibadan as the concentrations were below the level found in the control sample (*Table 1*).

### Total Lead and Cadmium Concentrations in Soil

The concentrations of lead and cadmium obtained in the residential settlements indicated possible contamination. Mean lead concentrations in the Lagos average income settlement (66.3 mg/kg), Lagos low income settlement (90.5 mg/kg), Ibadan average income settlement (77.7 mg/kg) and Ibadan low income settlement (63.1 mg/kg) had values which exceeded the Dutch limits of 50 mg/kg in residential soil (*Table 1*).^48^ The calculated I_geo_ values of the soil samples, which indicated the degree of anthropogenic pollution, revealed that average and low income settlements in Lagos and Ibadan had low levels of contamination with lead, while average and low income settlements in Lagos also had low levels of contamination with cadmium (*Table 2*). Lead showed a high CF value in the Lagos low income settlements (38.7), followed by the Ibadan average income settlements (33.2), while cadmium showed a high value in the Lagos average income and low income (2.07) settlements. Ibadan suburban settlements had the lowest CF value (0.22). The calculated CF values showed very high contamination of lead in the new high income, average and low income settlements of Lagos, and old and new high income, average income and low income settlements in Ibadan, while there was moderate cadmium contamination in all the settlements in Lagos, except for the suburban settlements (*Table 2*).

**Table 2:** Geo-accumulation Index (Igeo), Contamination Factor (CF) and Pollution Load Index (PLI) of Residential Settlements in Lagos and Ibadan, Nigeria

Settlement type	**I_geo_**		**CF**		**Total**	**PLI**
	Lead	Cadmium	Lead	Cadmium		
**Lagos**						
Lagos old high income	−2.34	0.66	2.54	1.80	4.34	1.54
Lagos new high income	−0.8	0.88	7.35	1.53	8.88	1.26
Lagos average income	1.41	1.08	28.3	2.07	30.4	1.97
Lagos low income	1.59	1.08	38.7	2.07	40.8	2.08
Lagos suburban	−1.21	−0.79	5.56	0.57	6.13	1.21
**Ibadan**						
Ibadan old high income	0.59	−0.73	19.3	0.74	20.0	1.52
Ibadan new high income	0.35	−0.4	16.4	0.58	17.0	1.50
Ibadan average income	1.37	−0.09	33.2	0.91	34.1	1.77
Ibadan low income	1.07	−0.21	27.0	0.85	27.9	1.68
Ibadan suburban	−0.36	−2.17	10	0.22	10.2	1.14

Note: Four categories adapted to describe the extent of contamination are CF <1: very low contamination, CF 1–3: low contamination, CF 3–6: moderate contamination and CF > 6: considerable contamination; I_geo_ values are categorized as <0: uncontaminated, 0–1: uncontaminated-moderately contaminated, 1.00–1.99: moderately contaminated, 2.00–2.99: more contaminated and 3.00–3.99: highly contaminated; a PLI value of one indicates only baseline levels of pollutants present and values above one indicate progressive contamination of the soil.

The pollution load index was used to assess the metal accumulation and multi-element contamination resulting in increased overall metal toxicity.^49^ The results of the pollution load index (*Table 2*) showed that there was accumulation of the metals in all the settlements in Lagos and Ibadan, while the Lagos low income settlement had the highest PLI (2.08). This indicates the potential accumulation of metals in the soils of all the residential settlements in both cities.

### Fractionation of Heavy Metals in the Soil Sample

Water soluble fraction (F1) represents those metals which are water soluble. Across the different settlements, the mean fractional content of lead was 0.80–8.30% in Lagos and 6.80–17.9% in Ibadan, while cadmium was 0% in all settlements. Metals in this fraction are assumed to be available for plant uptake.^50^ Absence of cadmium in this fraction suggests no current risk of cadmium contamination, but accumulation over time might result in possible health risks. Exchangeable fraction (F2) metals have high mobility and are readily available to plants and organisms. The mean fractional content of lead was 0.2–2.1% in Lagos and 2.6–4.9% in Ibadan, while that of cadmium was 0 – 10.3% in Lagos and 0.5–15.3% in Ibadan. Carbonate-bound fraction (F3) metals have medium mobility and are not readily available to plants. The mean fractional content of lead was 1.90–21.6% in Lagos and 15.4–27% in Ibadan, and the mean fractional content of cadmium was 3.40 - 44.6% in Lagos and 1.20–17.1% in Ibadan. Iron-manganese oxides-bound fraction (F4) metals under an extremely reducible condition can get into the environment from a contaminated site.^51^ The mean fractional content of lead was 10.4–93% in Lagos and 2.60–29.1% in Ibadan, while that of cadmium was 6.9 – 75.4% in Lagos and 36.7 – 59.3% in Ibadan. Organic-bound fraction (F5) metals have medium mobility and decomposition of the organic fraction occurs with time. The mean fractional content of lead was 0.5–22.9% in Lagos and 20.3–26.5% in Ibadan, while the mean fractional content of cadmium was 0–5.7% in Lagos and 0–12.5% in Ibadan. Residual fraction (F6) metals are crystal lattice fixed, mobilize slowly and decomposition of crystal can make them available.^52^ The mean fractional content of lead was 2.9–27% in Lagos and 17.1–28.3% in Ibadan and the fractional content of cadmium was 5.3–79.3% in Lagos and 18.6–42.8% in Ibadan (*Figure 2, Figure 3*).

### Metal Mobility Factors

The lead mobility factors were moderately high in the Lagos suburban settlement (44.3%) and low in the new high income (3.4%), old high income (5.3%), average income (10.6%) and low income (25.3%) settlements in Lagos. The mobility factors were low in the new high income (25.7%) and averagely high in the old high income (37%), suburban (33.8%), average income (45%) and low income (47%) settlements in Ibadan. The cadmium mobility factors were low in the old high income (7.6%), average income (16.1%), low income (19.3%) and suburban (13.7%) settlements; and moderately high in the new high income settlement (44.6%) in Lagos. All the settlements in Ibadan had low cadmium mobility factors in the new high income (2.4%), old high income (22%), and average income (17%) settlements, while the low income and suburban settlements had a mobility factor of 11.8% (*Table 3*). High values of mobility factors of heavy metals are evidence of relatively high bioavailability.^39^

**Table 3:** Mobility Factors of Lead and Cadmium in Residential Settlements in Lagos and Ibadan, Nigeria

**Settlement type**	**Lead (%)**	**Cadmium (%)**
**Lagos**		
Lagos new high income	3.40	44.6
Lagos old high income	5.30	7.60
Lagos average income	10.6	16.1
Lagos low income	25.3	19.3
Lagos suburban	44.3	13.8
**Ibadan**		
Ibadan new high income	25.7	2.40
Ibadan old high income	37.0	22.0
Ibadan average income	45.0	17.0
Ibadan low income	47.0	11.8
Ibadan suburban	33.8	11.8

### Statistical Analysis Results

Statistical analyses of the results obtained in the residential settlements in Lagos and Ibadan using analysis of variance (ANOVA) showed significant differences (p<0.05) by settlement type (*Table 4, Table 5*). In the Lagos settlements, there was a significant negative correlation between sand and silt, and sand and clay at p < 0.01, as well as a positive correlation at p < 0.05 between % soil organic matter and lead with r values of −0.895, −0.663 and 0.353, respectively (*Table 4*). In Ibadan, at p < 0.01, there was a significant positive correlation between % soil organic matter and cadmium (r = 0.506) and a negative correlation with pH (r = −0.410). In addition, pH showed a negative correlation with electrical conductivity (r = −0.296, p < 0.05). There was a strong negative correlation between sand and silt (r = −0.819, p < 0.01), sand and clay (r = −0.538, p < 0.01) and a positive correlation with electrical conductivity (r = 0.286, p < 0.05). There was a significant positive correlation between lead and cadmium (r = 0.299, p < 0.05) in Ibadan and Lagos (r = 0.230) (*Table 6, Table 7*).

**Table 4:** Analysis of Variance (ANOVA) Test of Difference in Mean Physiochemical Properties and Lead and Cadmium Concentrations in Lagos Residential Settlements (n=50)

**ANOVA**						
		Sum of Squares	df	Mean Square	F	Significance
pH	Between sites	1.576	4	.394	6.369	.000
	Within sites	2.784	45	.062		
	Total	4.360	49			
Soil organic matter	Between sites	2.989	4	.747	1.502	.218
	Within sites	22.385	45	.497		
	Total	25.373	49			
Electrical conductivity	Between sites	20034.417	4	5008.604	5.259	.001
	Within sites	42858.667	45	952.415		
	Total	62893.085	49			
Percentage sand	Between sites	103.520	4	25.880	4.742	.003
	Within sites	245.600	45	5.458		
	Total	349.120	49			
Percentage silt	Between sites	52.000	4	13.000	3.703	.011
	Within sites	158.000	45	3.511		
	Total	210.000	49			
Percentage clay	Between sites	9.120	4	2.280	1.574	.198
	Within sites	65.200	45	1.449		
	Total	74.320	49			
Lead	Between sites	56371.768	4	14092.942	4.260	.005
	Within sites	148879.314	45	3308.429		
	Total	205251.082	49			

**Table 5:** Analysis of Variance ANOVA Test of Differences in Mean Physiochemical Properties and Lead and Cadmium Concentrations in Ibadan Residential Settlements (n=50)

**ANOVA**						
		Sum of Squares	df	Mean Square	F Significance	
pH	Between sites	.839	4	.210	1.620	.186
	Within sites	5.828	45	.130		
	Total	6.667	49			
Soil organic matter	Between sites	13.955	4	3.489	2.709	.042
	Within sites	57.944	45	1.288		
	Total	71.899	49			
Electrical conductivity	Between sites	43911.798	4	10977.949	2.717	.041
	Within sites	181799.958	45	4039.999		
	Total	225711.756	49			
Percentage sand	Between sites	23.597	4	5.899	1.711	.164
	Within sites	155.184	45	3.449		
	Total	178.781	49			
Percentage silt	Between sites	13.280	4	3.320	1.339	.270
	Within sites	111.600	45	2.480		
	Total	124.880	49			
Percentage clay	Between sites	3.200	4	.800	.657	.625
	Within sites	54.800	45	1.218		
	Total	58.000	49			
Lead	Between sites	18016.843	4	4504.211	1.802	.145
	Within sites	112477.675	45	2499.504		
	Total	130494.518	49			
Cadmium	Between sites	.652	4	.163	1.716	.163
	Within sites	4.277	45	.095		
	Total	4.929	49			

**Table 6:** Correlation Coefficients of Soil Physiochemical Properties, Lead and Cadmium in Lagos Residential Settlements

		**Correlations**							
		pH	Soil organic matter	Electrical conductivity	Percentage sand	Percentage silt	Percentage clay	Lead	Cadmium
pH	Pearson correlation	1							
	Significance (2-tailed)								
Soil organic matter	Pearson correlation	−.056	1						
	Significance (2-tailed)	.700							
Electrical conductivity	Pearson correlation	−.226	−.050	1					
	Significance (2-tailed)	.114	.730						
Percentage sand	Pearson correlation	.195	.168	.202	1				
	Significance (2-tailed)	.175	.243	.159					
Percentage silt	Pearson correlation	−.238	−.084	−.193	−.895^**^	1			
	Significance (2-tailed)	.096	.562	.180	.000				
Percentage clay	Pearson correlation	−.022	−.224	−.114	−.663^**^	.259	1		
	Significance (2-tailed)	.878	.118	.431	.000	.069			
Lead	Pearson correlation	.020	.353^*^	.004	.047	−.025	−.060	1	
	Significance (2-tailed)	.893	.012	.976	.745	.862	.679		
Cadmium	Pearson correlation	−.027	.186	−.135	−.102	.147	−.027	.230	1
	Significance (2-tailed)	.852	.197	.352	.481	.307	.853	.109	

^*^. Correlation is significant at the 0.05 level (2-tailed).

^**^. Correlation is significant at the 0.01 level (2-tailed).

**Table 7:**
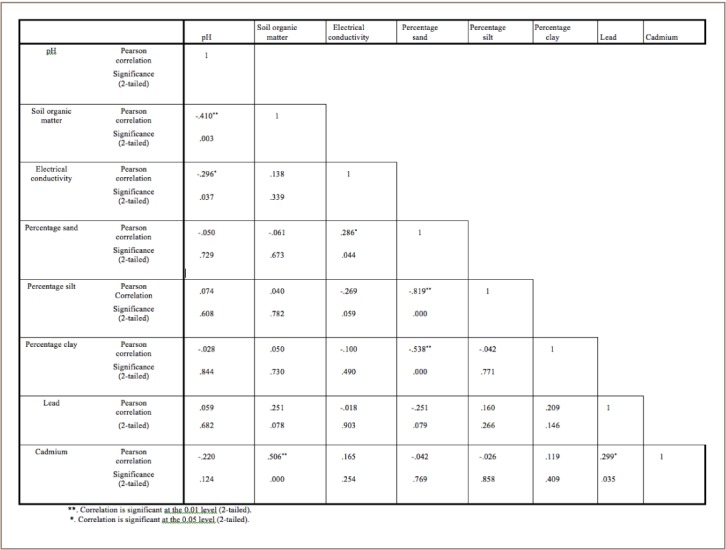
Correlation Coefficients of Soil Physicochemical Properties, Lead and Cadmium in Ibadan Residential Settlements

### Risk Assessment

The estimated BLLs of children between ages 0–7 years in the residential areas in Lagos and Ibadan using the US IEUBK model are presented in Table 8. The results showed that estimated BLL of children 0–7 years of age in all the residential settlements were lower than the BLL of concern (10 μg/dL) and reference level (5 μg/dL). The Lagos low income settlement had the highest probability density for children between 1–7 years of age with an estimated BLL of >10 μg/dL. This population made up less than 0.01% of those within this age range.

**Table 8:** Mean Estimated Blood Lead Levels (μg/dL) of Children in Residential Settlements in Lagos, Ibadan and the Control Soil Sample

Age of Children (years)	Settlements										

	Aa	Ab	Ac	Ad	Ae	Ba	Bb	Bc	Bd	Be	Ctr
0.5–1	1.0	1.1	1.6	1.9	1.1	1.4	1.3	1.8	1.6	1.2	0.9
1–2	1.0	1.2	1.8	2.1	1.1	1.5	1.4	2.0	1.8	1.2	1.0
2–3	0.9	1.1	1.7	2.0	1.0	1.4	1.3	1.8	1.6	1.2	0.9
3–4	0.9	1.0	1.6	1.9	1.0	1.3	1.3	1.7	1.5	1.1	0.9
4–5	0.8	0.9	1.4	1.6	0.9	1.2	1.1	1.5	1.3	1.0	0.8
5–6	0.8	0.9	1.2	1.4	0.8	1.1	1.0	1.3	1.2	0.9	0.8
6–7	0.7	0.8	1.1	1.3	0.8	1.0	0.9	1.2	1.1	0.8	0.7

Abbreviations: Aa, Lagos new high income settlement; Ab, Lagos old high income settlement; Ac, Lagos average income settlement; Ad, Lagos low income; Ae, Lagos suburban; Ba, Ibadan new high income settlement; Bb, Ibadan old high income settlement; Bc, Ibadan average income settlement; Bd, Ibadan low income settlement; Be, Ibadan suburban settlement; Ctr, Control settlement.

## Discussion

Soil contamination with heavy metals has severely increased globally due to human activities. Soil pollution and contamination with heavy metals can pose danger to human health. Soil contamination with heavy metals such as lead and cadmium has been recognized as a serious environmental and human health concern because they are non-biodegradable, have the tendency to accumulate in plants and animal tissues, including humans, and have been linked to chronic human diseases and death. Availability of heavy metals in soil is pH dependent.^53^,^54^ Most of the soil in the study areas had a pH between 6.0–9.0, where most metals do not usually occur in their free form, hence are not likely to be available for uptake by plants and microorganisms.^55^ The concentration of lead was greater than cadmium in all the residential topsoil samples. The order of lead and cadmium concentrations in the residential settlements was:

Lead: *Lagos:* Low income > average income > old high income > suburban > new high income
*Ibadan:* Average income > low income > new high income >old high income > suburban

Cadmium: *Lagos:* Low income = average income > old high income > new high income > suburban
*Ibadan:* Average income > low income > old high income > new high income > suburban

This trend could be attributed to the fact that the low income settlement was comprised of low earners with a relatively high population. The residents might not have the financial capability to paint their buildings regularly and some did not paint at all. The buildings might have been painted with paints which contained very high levels of lead and cadmium. Thus, in Lagos and Ibadan, low income and average income settlements had the highest concentrations of lead and cadmium, respectively. New high income settlements in Lagos had the lowest concentration of lead. All of the buildings in these areas were newly painted, and hence, had the lowest mean concentrations of lead in the topsoil. The suburban settlement had the lowest concentration of cadmium in both cities and lead in Ibadan. The suburban settlement was the poorest settlement and had a low population and a high level of illiteracy. Most of the residential buildings in this area were not painted, and thus had little lead and cadmium contamination of the topsoil. Heavy metal contamination is associated with a mixture of contaminants rather than just one metal contaminant.^54^ The higher the PLI, the more serious the heavy metal accumulation in the soil.^47^ All the residential settlements in Ibadan had a cadmium CF of less than 1 with low contamination of the topsoil, while with respect to lead, they had a very high contamination level, with a CF greater than 6. In most cases, the residential settlements in Lagos had a cadmium CF of 1–3, and thus were moderately contaminated with cadmium, except for the suburban settlement with a CF of 0.5, while the lead contamination factor in the settlements was greater than 6. Hence, progressive contamination of the topsoil of the residential settlements in both cities was evident based on the PLI.

The significant positive correlation between lead and cadmium in the residential soils indicates that they might have originated from a similar anthropogenic source.^43^ The estimated BLLs of children between ages 0–7 years in the residential areas in Lagos and Ibadan using the US IEUBK model might be attributed to residential building paints as the main source of lead exposure. Although there are multiple sources of lead exposure in homes, this study considered soil around the residential buildings and these results were used for the estimation. The results obtained suggested a low risk of elevated BLL in children between these ages in the study areas. Accumulation of lead over time might increase the exposed population and the level of exposure, which might pose a health concern. The fact that the BLLs in the study areas do not pose a high risk to children in these areas does not mean that the sale of paints with high levels of these toxic and hazardous metals should be encouraged and continued. However, it suggests that there was little or no risk of exposure to children in the areas from this potential source i.e., painting of the residential buildings, as shown by the results obtained from the US IEUBK model. In addition, children in these settlements and most parts of Nigeria are not routinely tested except in the northern part of the country, where lead poisoning has been reported.^33^ The Washington State Department of Health Expert Panel has identified a BLL between 5 and 9 μg/dL to be detrimental to children and follow up steps have been recommended at this level.^53^

## Conclusions

This study assessed the physicochemical parameters and levels of lead and cadmium and their forms in the soil of residential settlements in Lagos and Ibadan, Nigeria. Soil contamination was evaluated using the geo-accumulation index, contamination factor and pollution load index. The risk of exposure to children between 0–7 years of age were also estimated using the US IEUBK model for lead in blood. The results obtained showed that there were varying concentrations of lead and cadmium in all of the sampled residential settlements in Lagos and Ibadan. Average and low income settlements in both cities had mean lead concentrations above the Dutch permissible limits in residential soil. In addition, the mean concentrations of the metals were above the mean concentrations found in the control sample, which suggested contamination of the soil.^48^ Very high contamination of the soil was observed as shown by the contamination factor and pollution load index, which might be attributed to painting of the residential buildings in the study areas. Painting of buildings may be one of the potential sources of heavy metal loading of the environment. Therefore, the production, sales and use of paints with high levels of heavy metals such as lead and cadmium should be discouraged and discontinued. There is an urgent need for regulations of heavy metals in consumer products such as paints in Nigeria to prevent the human health risks associated with exposure of vulnerable groups such as children to metals in these products and soil through ingestion, dust inhalation and dermal contact. Multiple sources of lead in homes should be identified and assessed to determine the level of exposure of vulnerable groups, especially children, and routine monitoring of children's BLLs is highly recommended in Nigeria.
